# *Leishmania infantum* Exoproducts Inhibit Human Invariant NKT Cell Expansion and Activation

**DOI:** 10.3389/fimmu.2017.00710

**Published:** 2017-06-19

**Authors:** Renata Belo, Nuno Santarém, Cátia Pereira, Begoña Pérez-Cabezas, Fátima Macedo, Maria Leite-de-Moraes, Anabela Cordeiro-da-Silva

**Affiliations:** ^1^Parasite Disease Group, Institute for Molecular and Cell Biology (IBMC), Institute for Investigation and Innovation in Health (i3S), Porto, Portugal; ^2^Laboratory of Immunoregulation and Immunopathology, Institut Necker-Enfants Malades, CNRS UMR 8253 and INSERM UMR 1151, Paris, France; ^3^Université Paris Descartes Sorbonne Paris Cité, Paris, France; ^4^Cell Activation and Gene Expression, Institute for Molecular and Cell Biology (IBMC), Institute for Investigation and Innovation in Health (i3S), Porto, Portugal; ^5^Department of Medical Science, Aveiro University, Aveiro, Portugal; ^6^Faculty of Pharmacy, Department of Biological Sciences, University of Porto, Porto, Portugal

**Keywords:** *Leishmania*, NKT cells, exoproducts, peripheral blood mononuclear cell, parasite escape

## Abstract

*Leishmania infantum* is one of the major parasite species associated with visceral leishmaniasis, a severe form of the disease that can become lethal if untreated. This obligate intracellular parasite has developed diverse strategies to escape the host immune response, such as exoproducts (Exo) carrying a wide range of molecules, including parasite virulence factors, which are potentially implicated in early stages of infection. Herein, we report that *L. infantum* Exo and its two fractions composed of extracellular vesicles (EVs) and vesicle-depleted-exoproducts (VDEs) inhibit human peripheral blood invariant natural killer T (iNKT) cell expansion in response to their specific ligand, the glycolipid α-GalactosylCeramide (α-GalCer), as well as their capacity to promptly produce IL-4 and IFNγ. Using plate-bound CD1d and α-GalCer, we found that Exo, EV, and VDE fractions reduced iNKT cell activation in a dose-dependent manner, suggesting that they prevented α-GalCer presentation by CD1d molecules. This direct effect on CD1d was confirmed by the observation that CD1d:α-GalCer complex formation was impaired in the presence of Exo, EV, and VDE fractions. Furthermore, lipid extracts from the three compounds mimicked the inhibition of iNKT cell activation. These lipid components of *L. infantum* exoproducts, including EV and VDE fractions, might compete for CD1-binding sites, thus blocking iNKT cell activation. Overall, our results provide evidence for a novel strategy through which *L. infantum* can evade immune responses of mammalian host cells by preventing iNKT lymphocytes from recognizing glycolipids in a TCR-dependent manner.

## Introduction

Leishmaniasis is a neglected tropical disease caused by parasites of the genus *Leishmania*. These parasites have a complex digenetic life cycle, adopting an extracellular, flagellated, and motile form (promastigotes) in the insect vector and an intracellular, non-motile form (amastigotes) in the vertebrate host (1–3). Promastigotes, inside the mammalian host, infect phagocytic cells where they rapidly transform into the amastigote form that replicates, ruptures the host cell, and spreads the infection. The clinical manifestations of the disease vary with parasite species and host immune responses, ranging from subclinical infection, and self-healing lesions to chronic symptoms that can ultimately lead to death (1–3). A successful infection depends on the different mechanisms of evasion developed by the parasite, enabling it to subvert the immune responses and to persist in the mammalian host (4, 5). The main strategy consists in inducing antigenic diversion, which impairs both innate and adaptive immune responses (4, 5).

Recent research on new mechanisms of parasite escape has emphasized the capacity of *Leishmania* to release exoproducts (Exo) (6–8). We have shown that Exo can be separated into two fractions, namely vesicle-depleted-exoproducts (VDEs) and extracellular vesicles (EVs) (6). The latter, which are released into the extracellular environment by many types of eukaryotic and prokaryotic cells, are currently the focus of much interest. They are delimited by a lipid bilayer including specific proteins, lipids, and mRNA, depending on their cellular origin (9–12). These vesicles have been extensively studied because of their capacity to mediate intercellular communication that does not require cell-to-cell contact (9–12). In the context of infections by parasites, such as *L. infantum*, the influence of total Exo, EV, and VDE fractions on mammalian host immune responses is still a matter of debate.

Invariant natural killer T (iNKT) cells form a distinct population of T lymphocytes with innate-like properties. They express an invariant TCR α-chain (Vα14-Jα18 in mice and Vα24-Jα18 in humans) associated with a restricted set of Vβ chains, including Vβ8, Vβ7, and Vβ2 in mice and Vβ11 in humans. Unlike conventional T cells, which recognize peptide antigens in the context of MHC class I or II molecules, iNKT cells are restricted by glycolipid antigens presented by the non-polymorphic MHC class I-like molecule CD1d (13–16). The α-galactosylceramide (α-GalCer) is a potent and specific antigen that is frequently used in the study of iNKT cells for stimulation and identification by the use of α-GalCer-loaded CD1d tetramers (17). The innate-like character of iNKT cells is revealed by their ability to rapidly respond to stimulation by producing a wide range of cytokines, such as IFNγ, IL-4, IL-17, IL-10, and IL-13, which endows these cells with an exceptional immunomodulatory potential (18–21). iNKT cells are implicated in a number of immune responses including autoimmunity, tumor immunology, and immunity against viral, bacterial, fungal, and parasitic pathogens, namely *Leishmania* (22–29). Additionally, iNKT cells play a major role in patrolling the body and in mounting distinct immune responses to infections (30, 31).

Here, we found that exposure to Exo, EV, and VDE fractions led to the inhibition of human peripheral blood iNKT cell expansion and cytokine production in response to α-GalCer. Starting from this finding, we set out to examine the mechanisms through which this inhibition occurred and attempted to identify the molecules involved as well as their mode of action.

## Materials and Methods

### Parasites

A cloned line of virulent *Leishmania infantum* (MHOM/MA/67/ITMAP-263) was maintained by weekly sub-passages at 26°C in RPMI 1640 medium supplemented with 10% heat-inactivated fetal bovine serum (FBS), 100 U/ml penicillin, 100 mg/ml streptomycin, and 20 mM HEPES (all from Lonza). Only promastigotes from up to 10 passages were used in the experiments. Before recovery of extracellular material, parasites were transferred to cRPMI, a protein-deprived medium composed of RPMI base supplemented with SDM base and hemin, which was previously optimized for exosome studies (32). The starting inoculum for all cultures was 1 × 10^6^ parasites/ml.

### Preparation of *L. infantum* Extracellular

Promastigotes were grown in cRPMI for 4 days as previously described (32). Parasites were removed from culture supernatant by centrifugation and filtration through a 0.4 µm filter. To recover the total exoproducts, the filtrated supernatant was concentrated in a centrifugal filter unit with a membrane nominal molecular weight limit (NMWL) of 3 kDa. To isolate EV, the filtrated supernatant was concentrated in a centrifugal filter unit with a NMWL of 100 kDa and ultracentrifuged overnight at 100,000 *g*, 4°C. The volume that passed through was further concentrated in a centrifugal filter unit with a NMWL of 3 kDa, in order to obtain VDE. These three preparations were dialyzed twice against PBS using the respective filter devices and resuspended in PBS. Finally, each preparation was passed through a 0.2 µm filter. Note that 1× Exo, EV, or VDE designates products released from 1 × 10^7^ parasites per microliters (Figure S1 in Supplementary Material).

We used EV isolated from the human pancreas adenocarcinoma-derived BxPC-3 cell line as a negative control (33) (hereafter termed heterologous EV or hEV). Heterologous EVs were prepared following the ExoQuick-TC™ instruction manual. Briefly, the supernatant of the BxPC-3 cell line, previously cultured in RPMI medium with exosome-free FBS, was collected and centrifuged 15 min at 3,000 *g* to remove cells and debris. The supernatant was then incubated overnight with the ExoQuick-TC™ precipitation solution at 4°C. The preparation was centrifuged at 1,500 *g* for 30 min., supernatant was discarded, and the hEVs were resuspended in PBS.

### Biological Samples

Peripheral blood mononuclear cells were collected from healthy donors at the Etablissement Français du Sang (EFS), Paris, France, and at the Immuno-haemotherapy Department of Hospital de São João, Porto, Portugal. Experiments were performed in accordance with the Helsinki Declaration, with informed consent received from each donor and managed by each institution. PBMCs were isolated by density-gradient centrifugation (Ficoll-Paque PLUS, GE Healthcare or Histopaque-1077, Sigma). C57BL/6J mice used for the preparation of bone marrow-derived dendritic cells (BM-DCs) were maintained in the animal facility at the Instituto de Biologia Molecular e Celular (IBMC, Porto, Portugal). Animals were euthanized in accordance with the IBMC.INEB Animal Ethics Committees and the Portuguese National Authorities for Animal Health guidelines (directive 2010/63/EU).

### Human iNKT Cell Expansion

Peripheral blood mononuclear cells were cultured in 24-well plates at a density of 10^6^ cells per well in RPMI 1640 medium containing antibiotics, 10% heat-inactivated FBS, 200 mM glutamine, and 10 mM HEPES (all from Invitrogen or Lonza) with 100 ng/ml α-GalCer (Alexis-Coger SA), as previously described (18, 19). When indicated, *L. infantum* Exo, EV, VDE, and hEV were added at the onset of culture. Twenty-four hours later, rhIL-2 (purchased from R&D or kindly provided by the National Cancer Institute) was added. After 8–12 days, cells were harvested, extensively washed, and counted using trypan blue dye exclusion for dead cells. Cells were further stained and analyzed by flow cytometry.

### Surface and Intracellular Staining

Fresh or cultured PBMCs were stained and analyzed by flow cytometry as previously described (18, 19). Surface staining was performed in PBS buffer containing 2% FBS and 0.01% NaN3, using PBS57-loaded-CD1d-tetramers (from the National Institutes of Health Tetramer Core Facility) (in all Figures stated as CD1d-tetramers), and the following directly conjugated anti-human monoclonal antibodies: PerCP-Cy5.5-labeled anti-CD3 (OKT3), PE-Cy7-labeled anti-CD4 (RPA-T4), and antigen-presenting cell (APC)-labeled-anti-CD8 (RPA-T8) (eBioscience).

For intracellular cytokine staining, CD1d-transfected C1R cells were incubated with α-GalCer alone or in the presence of *L. infantum* Exo, EV, and VDE. Human iNKT cell lines and 10 µg/ml brefeldin A (Sigma-Aldrich) were added 2 h later. After a 5-h incubation, cells were harvested and washed before fixation with 4% paraformaldehyde and permeabilization using 1% saponin (Sigma-Aldrich). Cells were then stained with anti-CD3, APC-labeled anti-IL-4 (8D4-8), and PE-Cy7-anti-IFNγ (4S.B3) (eBioscience). Data were acquired on a FACS Canto II flow cytometer (BD Biosciences) and analyzed with FlowJo software (10.2).

### Detection of CD1d:α-GalCer Complex Formation

Dendritic cells (DCs) were derived from bone marrow (BM) precursors as previously described (34). In brief, BM precursors recovered by flushing femurs and tibias from the hind legs of C57BL/6J mice were suspended in RPMI supplemented with 10% heat-inactivated FBS, 100 U/ml penicillin, 100 µg/ml streptomycin, and 10 mM HEPES (all from Lonza), 50 µM 2-Mercaptoethanol (Sigma Chemical), and 10% of J558 cell line supernatant containing GM-CSF (J558-GM-CSF) and cultured at 37°C with 5% CO_2_ in T75 culture flasks with a vented cap. At day 3, the same amount of supplemented medium was added to each flask. At day 6 and 8, half of the volume of each flask was recovered and centrifuged and the cell pellet was resuspended in the same amount of supplemented medium. At day 9, BM-DCs were harvested, washed, and cultured in RPMI 2% FBS with 100 ng/ml α-GalCer and/or increasing doses (1, 10, 20×) of *L. infantum* Exo, EV, or VDE for 4 h. CD1d expression and formation of CD1d:α-GalCer complexes on the surface of BM-DC was then determined by flow cytometry using the following mAbs: anti-mouse CD1d (51.1) (eBioscience) and anti-mouse CD1d:α-GalCer (L363, Biolegend).

### Human iNKT-Cell Line Generation and Restimulation

A polyclonal primary iNKT cell line was generated as described (35) according to a previously established protocol (36). In brief, PBMCs from healthy donors were cultured with 100 ng/ml of α-GalCer (KRN7000) and 100 U/ml of IL-2 (R&D Systems). After 11 days, CD1d-PBS57 tetramer^+^CD3^+^ cells were sorted using a FACSAria cell sorter (BD Biosciences). When cells stopped proliferating, 1 µg/ml of PHA (Thermo Fisher Scientific, Waltham, MA, USA) was added together with irradiated PBMC for restimulation.

### iNKT Cell Activation Assays

Invariant natural killer T cell activation assays were performed using CD1d-transfected C1R cells as APCs or soluble mouse CD1d coated in 384-well plates.

For assays using APC, CD1d-transfected C1R cells were cultured for 4 h with 5 or 25 ng/ml of α-GalCer alone or together with increasing doses (1, 10, 20×) of *L. infantum* Exo, EV, or VDE. The human polyclonal primary iNKT cell line was then added and supernatants from this co-culture were harvest 40 h later and assayed for GM-CSF by ELISA using purified (BVD2-23B6) and biotinylated (BVD2-21C11) anti-GM-CSF mAbs (Biolegend). CD1d (NIH tetramer core facility) was immobilized on 384-well Maxisorp treated microplates (Nunc, Rochester, NY, USA). After overnight incubation at 37°C, a mixture of 25 or 100 ng/ml α-GalCer and *L. infantum* Exo, EV, VDE, or hEV was added for 24 h. In some experiments, α-GalCer (25 ng/ml) was added 8 h before or after Exo, VDE, or EV (20×). After extensive washing, the 24.8 iNKT cell hybridoma (25,000/well) was added for further 20 h. Culture supernatants were recovered, and IL-2 concentrations were determined by ELISA using purified (JES6-1A12) and biotinylated (JES6-5H4) anti-IL-2 mAbs (Biolegend).

### Lipid Extraction and Quantification

Total lipid content was isolated from Exo, EV, and VDE using the Bligh and Dyer method (37). The lower organic phase was dried under nitrogen at 40°C and samples were kept at −20°C until quantification. Total lipid content of each sample was assessed by the sulpho-phospho-vanillin colorimetric assay. Lipid extracts were solubilized in chloroform and incubated at 90°C in a heater block until solvent evaporation. Increasing concentrations of cholesterol (Sigma-Aldrich) dissolved in chloroform were treated similarly and used as standard. Upon addition of 96% sulfuric acid samples were further incubated at 90°C for 20 min and then cooled at 4°C for 4 min followed by the addition of 0.2 mg/ml vanillin in 17% phosphoric acid. The samples were then incubated at room temperature for 10 min and absorbance was measured at 540 nm. They were finally re-dried and resuspended in RPMI 10% FBS at appropriate concentrations to be used in functional assays. To facilitate solubilizing of lipid extracts in aqueous medium, the samples were dissolved first in a minimum volume of methanol.

### Statistical Analysis

Values are expressed as the mean ± SEM, and significant differences were determined using either Mann–Whitney non-parametric *t*-tests or ANOVA with 95% confidence interval (GraphPad Prism 6). *p*-Values (*p* < 0.05) were considered significant. *p < 0.05, **p < 0.01, ***p < 0.001.

## Results

### *L. infantum* Exoproducts Inhibit iNKT Cell Expansion

**Figure 1:**
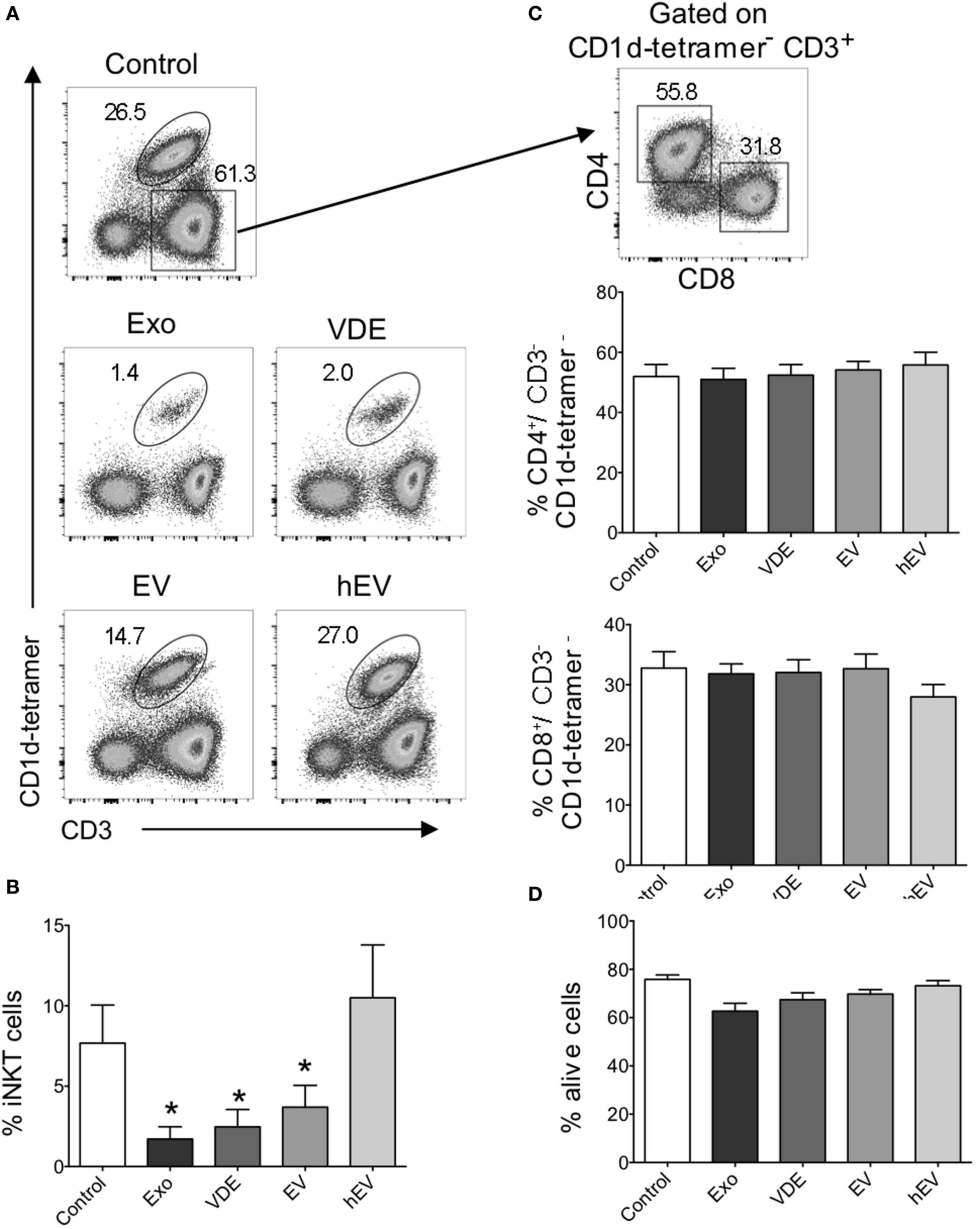
Invariant natural killer T (iNKT) cell expansion was impaired in the presence of *Leishmania infantum* Exo, extracellular vesicle (EV), and vesicle-depleted-exoproducts (VDEs). **(A)** Representative FACS profile showing the gating strategy used to identify iNKT cells following 8–12 days of culture with α-GalCer alone (control) or together with *L. infantum* Exo, EV, VDE, or hEV. iNKT cells were identified as CD3^+^ CD1d-tetramer^+^ cells. **(B)** Histograms represent the percentage of iNKT cells, as described in **(A)**. **(C)** Representative FACS profile showing the gating strategy used to identify CD4^+^ and CD8^+^ conventional T cells among CD3^+^CD1d tetramer^−^ peripheral blood mononuclear cell (PBMC) after 8–12 days of culture in the presence of α-GalCer. Histograms represent the percentage of CD4^+^ and CD8^+^ T cells among CD3^+^CD1d tetramer^−^ cells. **(D)**. Histograms represent the percentage of living cells, detected by 7AAD staining, among whole PBMC cultured in the presence of α-GalCer. Data are shown as means ± SEM (*n* = 14–24 individual donors tested). All groups were tested versus control group. *p < 0.05.

Previous reports support a possible influence of iNKT cells on the outcome of experimental *Leishmania* infection (38, 39). However, there are only few data documenting their impact on the severity of the human pathology. Our expertise in preparing exoproducts as well as EV and VDE fractions (6), together with the human iNKT cell expansion protocol currently used in our laboratory (18, 19), enabled us to examine how *L. infantum* Exo affected the functional properties of these lymphocytes. iNKT cells were identified by their expression of CD3 and CD1d-tetramers loaded with PBS57 antigen (α-GalCer analog), as previously described (18, 19). PBMCs isolated from healthy donors were cultured with α-GalCer alone or together with *L. infantum* Exo, EV, VDE, or heterologous EV (hEV) used as a negative control. Exo, as well as VDE and EV significantly impaired the expansion of iNKT cells (Figures 1A,B), while unrelated hEV had no effect (Figures 1A,B). *L. infantum* Exo, EV, and VDE targeted iNKT cells specifically since the frequency of conventional CD4^+^ and CD8^+^ T cells was not modified under these culture conditions (Figure 1C). Moreover, a possible toxicity of *L. infantum* extracts could be ruled out because total cell viability was not impaired (Figure 1D).

These findings clearly establish that *L. infantum* Exo, EV, and VDE fractions inhibit human peripheral blood iNKT cell expansion without affecting the frequency of conventional CD4^+^ and CD8^+^ T lymphocytes.

### *L. infantum* Exoproducts Impair Cytokine Production by iNKT Cells

Invariant Natural Killer T cells are known for their ability to promptly and massively secrete cytokines when stimulated.To investigate how *L. infantum* Exo, EV, and VDE fractions affected this biological activity, we took advantage of a human polyclonal primary iNKT cell line instead of the heterogeneous *in vitro* expanded PBMC population. These cells were co-cultured with CD1d-transfected C1R cells loaded with α-GalCer in the presence of increasing concentrations (1, 10, and 20×) of *L. infantum* extracellular products. iNKT cell activation was assessed 40 h later. We validated that non-transfected C1R cells were unable to stimulate iNKT cells in these conditions (data not shown).

**Figure 2:**
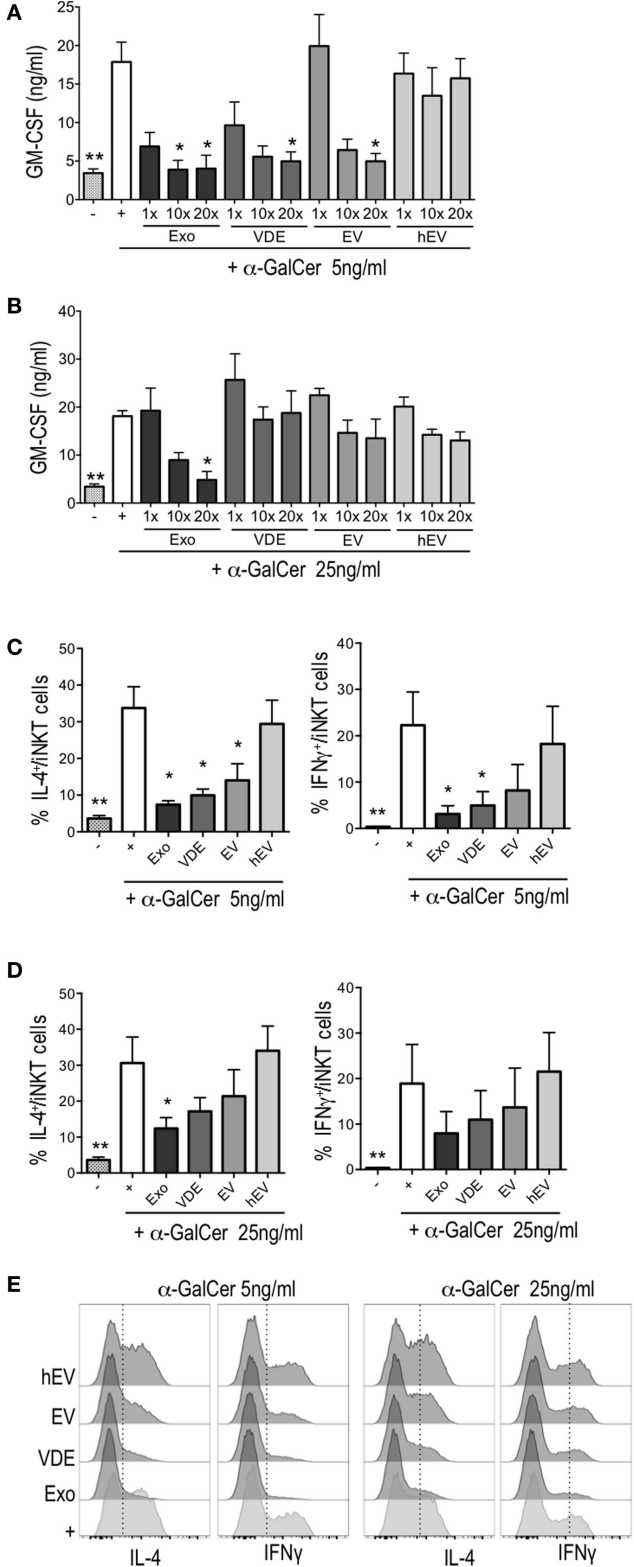
*Leishmania infantum* Exo, extracellular vesicle (EV), and vesicle-depleted-exoproduct (VDE) altered invariant natural killer T (iNKT) cell function. The polyclonal human iNKT cell line was incubated with CD1d-transfected C1R cells alone (−) or previously loaded with α-GalCer 5 ng/ml **(A,C)** or 25 ng/ml **(B,D)** with (+) or without *L. infantum* Exo, EV, VDE, or hEV. iNKT cell activation was measured by assessing GM-CSF concentrations in culture supernatants **(A,B)** and by determining the percentage of IL-4^+^ or IFNγ^+^ cells among the iNKT cell line **(C–E)**. Data show means ± SEM and the results are representative of two to three independent experiments (*n* = 4). All groups were tested versus the positive α-GalCer (+) control group. *p < 0.05, **p < 0.01. **(E)** Representative FACS profile of the results presented at **(C,D)**.

First, we ruled out a possible effect of Exo, EV, and VDE fractions *per se* on iNKT by evaluating the secretion of GM-CSF that did clearly not take place in response to these compounds (Figure S2 in Supplementary Material). However, all three exoproducts inhibited the activity induced by α-GalCer in a dose-dependent manner (Figure 2A). The dose of 20× was the most effective for all compounds. Of note, a fivefold increase in α-GalCer concentration reversed this inhibition, except for the dose of Exo 20× (Figure 2B). hEV had no significant effect on iNKT cell activation, whatever the dose (Figures 2A,B).

Next, we tested the ability of *L. infantum* exoproducts to modulate the production of cytokines by iNKT cells exposed for a short period to α-GalCer. To this end, CD1d-transfected C1R cells were loaded for 2 h with α-GalCer with or without Exo, EV, and VDE fractions at the dose of 20×. After extensive washings, iNKT cells were added to the culture for 5 h. All three compounds inhibited IL-4 and IFNγ production in response to α-GalCer (5 ng/ml), as shown in Figures 2C,E. The inhibition of IFN-γ secretion by EV was less pronounced, while Exo and VDE remained significant throughout (Figures 2C,E). Higher doses of α-GalCer (25 ng/ml) restored the production of IL-4 by iNKT cells partially and that of IFNγ completely (Figures 2D,E). hEV had no effect whatsoever whether α-GalCer was used at 5 or 25 ng/ml (Figures 2C–E). Exo, EV, and VDE fractions were unable to stimulate IFNγ and IL-4 production by iNKT cells when incubated with CD1d-transfected C1R cells in the absence of α-GalCer (Figure S3 in Supplementary Material).

Overall, these results suggest that Exo, EV, and VDE fractions might prevent optimal presentation of α-GalCer by CD1d molecules either directly by competing for α-GalCer-binding sites or indirectly by downregulating CD1d expression by transfected C1R cells.

### *L. infantum* Exo, EV, and VDE Fractions Exert a Direct Effect on CD1d Binding

**Figure 3:**
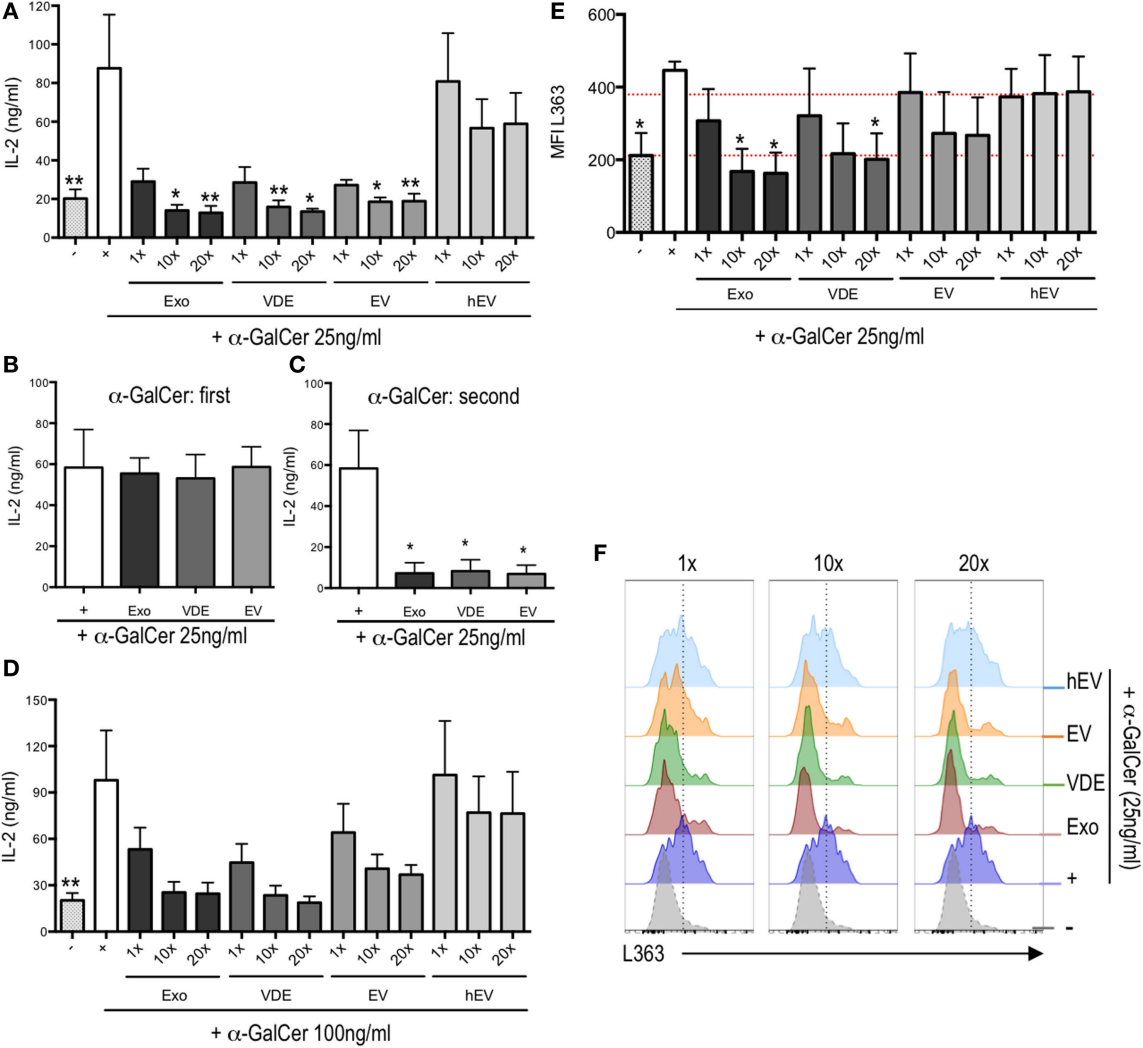
*Leishmania infantum* Exo, extracellular vesicle (EV), and vesicle-depleted-exoproduct (VDE) inhibited invariant natural killer T (iNKT) cell activation by plate-bound α-GalCer/CD1d complexes in a dose-dependent manner. Plate-bound mouse CD1d was loaded with a mixture of α-GalCer 25 ng/ml **(A,E,F)** and 100 ng/ml **(D)** alone (+) or in the presence of *L. infantum* Exo, EV, VDE, or hEV at different doses. **(B)** Plate-bound mouse CD1d was loaded first with α-GalCer at 25 ng/ml. Eight hours later, *L. infantum* Exo, EV, VDE, or hEV (20×) was added. **(C)** Plate-bound mouse CD1d was loaded with *L. infantum* Exo, EV, VDE, or hEV (20×). Eight hours later, 25 ng/ml of α-GalCer was added (second). After extensive washings, the 24.8 iNKT cell hybridoma was added and supernatants were recovered 20 h later. Data are expressed as means ± SEM of IL-2 levels detected in culture supernatants. Data are representative of two independent experiments (*n* = 4). All groups were tested versus positive α-GalCer (+) control group. *p < 0.05, **p < 0.01. **(E)** Histograms indicating MFI of L363 staining showing CD1d:α-GalCer complexes expressed by bone marrow-derived dendritic cells unstimulated (−) or previously incubated with α-GalCer (+) or α-GalCer plus *L. infantum* Exo, EV, VDE, or hEV at distinct doses. Data are expressed as means ± SEM from three independent experiments. **(F)** Representative FACS profile showing the expression of L363 as described in **(E)**. Data are representative of three independent experiments.

To asses whether *L. infantum* exoproducts inhibited α-GalCer binding to CD1d molecules directly, we used a cell-free system of stimulation in which plate-bound mCD1d were loaded with α-GalCer with or without increasing doses (1, 10, and 20×) of Exo, EV, and VDE fractions. Following extensive washings, we added 24.8 iNKT hybridoma cells and assessed their activation by measuring IL-2 levels in supernatants. As shown in Figure 3A, IL-2 secretion in response to 25 ng/ml α-GalCer was significantly inhibited by all three fractions at doses of 10× and 20×. Note that the inhibition was completely abrogated when α-GalCer was added before (Figure 3B), and not after (Figure 3C), *L. infantum* Exo, EV, and VDE fractions. A similar reversal occurred when α-GalCer was used at a fourfold higher dose (Figure 3D). In none of these conditions, did hEV modify IL-2 production by α-GalCer-stimulated iNKT cells (Figures 3A,D), which was also not altered in response to *L. infantum* Exo, EV, and VDE fractions *per se* (Figure S4A in Supplementary Material).

To further strengthen these results, we used the L363 mAb, which specifically recognizes CD1d:α-GalCer complexes to quantify these interactions (40). We found a clear inhibition of complex formation in response to increasing doses of *L. infantum* Exo and VDE fractions (Figures 3E,F). Note that exoproducts did not alter the expression of CD1d by BM-DC, which further excludes an indirect effect *via* receptor downmodulation (Figure S4B in Supplementary Material).

Overall, these findings indicate that *L. infantum* Exo, EV, and VDE fractions directly block CD1d:α-GalCer complex formation. Furthermore, experiments with plate-bound CD1d do not support an indirect mechanism involving CD1d downregulation.

### The Inhibition of iNKT Cell Activation by *L. infantum* Secreted Antigens Is Mediated through Lipids

**Figure 4:**
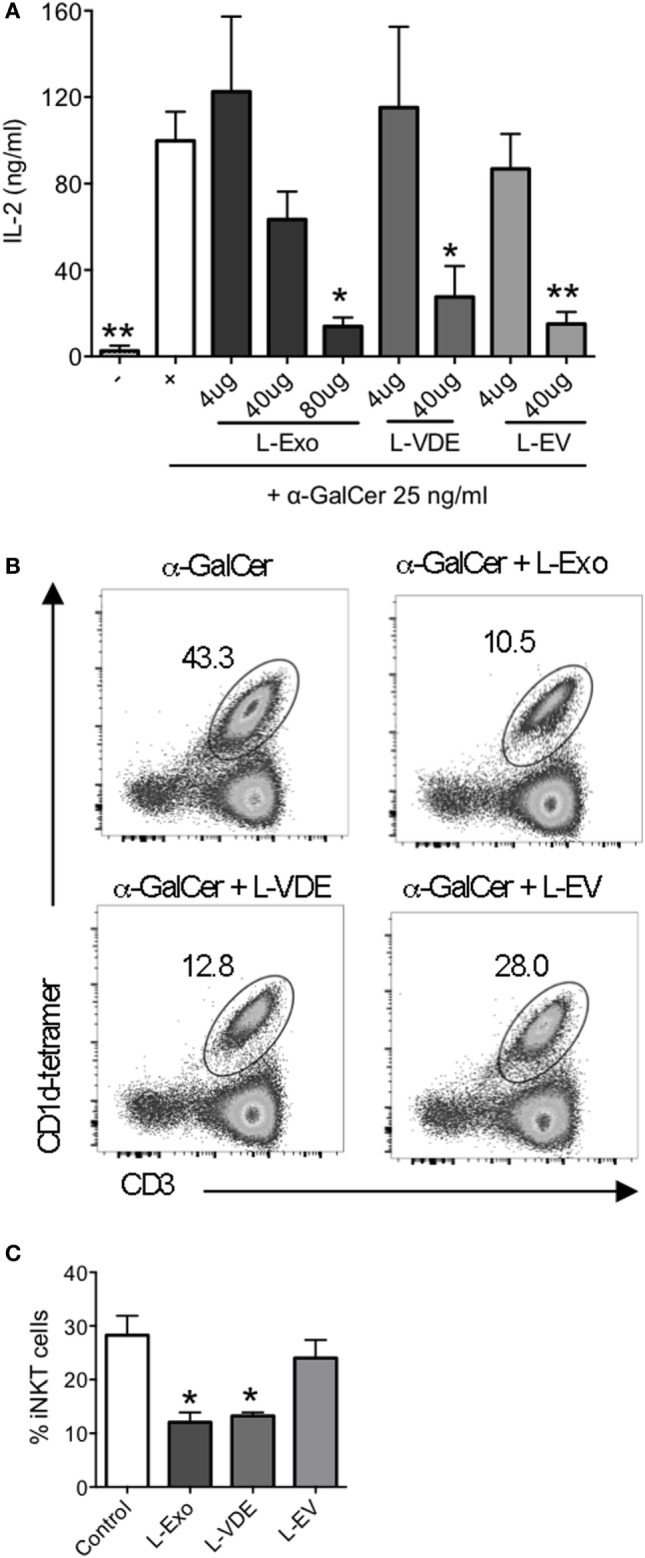
Lipids from *Leishmania infantum* Exo, extracellular vesicle (EV), and vesicle-depleted-exoproduct (VDE) mimicked the inhibition of invariant natural killer T (iNKT) cell activation. **(A)** Plate-bound mouse CD1d was loaded with α-GalCer (25 ng/ml) solely or in the presence of distinct doses of lipid extracts from *L. infantum* Exo, EV, VDE. 24.8 iNKT cell hybridoma was added and IL-2 production in the supernatants was measured. Data are expressed as means ± SEM of IL-2 concentrations in culture supernatants. Data are representative of two independent experiments (*n* = 4). All groups were tested versus the positive α-GalCer (+) control group. *p < 0.05, **p < 0.01. **(B)** Representative FACS profile showing the percentage of iNKT cells obtained after culture of peripheral blood mononuclear cell with α-GalCer in the presence of total lipid extracts from *L. infantum* Exo, EV, VDE. **(C)** Histograms represent the percentage of iNKT cells, as described in **(B)**. Data are expressed as means ± SEM and they are representative of two to five independent experiments.

In the light of the above results, we addressed the question of the identity of the molecules that blocked CD1d:α-GalCer complex formation. Knowing that glycolipids have been identified as the major CD1d ligands (15) and that the surface of *L. infantum* is covered by a glycocalix (41), it was tempting to hypothesize that the lipids present in Exo, VE, and EV preparation were responsible for the inhibition of iNKT cell activation. Hence, we extracted total lipids from Exo, EV, and VDE fractions and tested them in the plate-bound CD1d assay. Figure 4A shows that at high doses, they were all capable of inhibiting iNKT cell activation.

As a proof of concept, we tested whether the lipid extracts could also inhibit iNKT cell expansion from total PBMC, which was actually the case, as depicted in Figures 4B,C.

Collectively, these data demonstrate that lipids present in *L. infantum* Exo, EV, and VDE fractions account for the inhibition of iNKT cell activation induced by whole fractions.

## Discussion

Leishmaniasis remains a major public health issue because an effective human vaccine has not been developed so far. The most likely reason for this failure is that *Leishmania* spp. uses a variety of strategies to divert the immune system. Herein, we provide evidence for a new mechanism of evasion, which causes inhibition of TCR-dependent expansion and activation of human iNKT cells through release of exoproducts (Exo), EVs, and VDE by *L. infantum* promastigotes.

It is now widely acknowledged that Exo and its EV fraction are important players in the horizontal transfer of information between different cells, without direct contact. In this context, EVs could act as messengers capable of priming host cells to facilitate parasite infections (42). For instance, *L. infantum* promastigotes constitutively secrete EVs in the lumen of sandfly midgut, which contribute to the initial infective inoculum, being co-ingested with the parasite during the bite that initiates the pathology (43). *Leishmania*-derived EVs can ensure the transport of parasite virulence factors, such as GP63 proteins, to host cells. GP63 has previously been shown to interfere with host cell signaling pathways by inducing cleavage and activation of protein tyrosine phosphatases, downregulation of pro-inflammatory transcription factors and kinases, and decrease of NO production, thus weakening antimicrobial mechanisms of defense (6, 8, 44). It has also been reported recently that *Leishmania* exosomes could become immunosuppressive by modulating monocyte cytokine responses to IFNγ (45).

The impact of *Leishmania* Exo on immunoregulatory T lymphocytes, including iNKT cells, remains to be determined. The high degree of conservation between murine and human iNKT cell TCRs allows the same antigens presented by the non-polymorphic class-I-like molecule CD1d, such as α-GalCer, to be recognized by both species, which strongly supports an important biological function. iNKT cells can be activated by two distinct TCR-dependent pathways, namely recognition of foreign (direct way) or self (indirect way) lipid agonists (46). The latter are induced during cell stress following injury or infection. Several microbes contain lipids that can be presented by CD1d molecules and directly activate iNKT cells. Examples are glycosphingolipids with α-linked glucuronic or galacturonic acid in *Sphingomonas* species (47) and diacylglycerols with α-linked glucosyl or galactosyl moieties in *Borrelia burgdorferi* (35) and *Streptococcus pneumoniae* (48). In addition to this pathogen, antigen-dependent mechanism of activation, microbes can also use indirect pathways to activate iNKT cells. For instance, a previous report (27) has shown that *L. infantum*-infected DCs can upregulate their CD1d expression, thus becoming a target efficiently recognized and killed by activated IFNγ-producing iNKT cells. Furthermore, *Leishmania mexicana* lipophosphoglycan (LPG) (49) stimulates DCs through an indirect pathway to produce IL-12 and IL-18 that, in turn, can induce IFN-γ production by iNKT cells (50). Our findings indicate that *L. infantum* developed a means to evade protective immune responses by releasing Exo, EV, or VDE compounds to restrain the expansion and antigen activation of human iNKT cells.

Using plate-bound CD1d and L363 antibody to detect the formation of CD1d:α-GalCer complexes, we established that *L. infantum* Exo, VDE, and EV impaired iNKT cell activation by preventing the binding of α-GalCer to CD1d molecules. This effect was abolished at higher doses or when α-GalCer was coated before the addition of *L. infantum* exoproducts revealing a potential competition between these compounds and α-GalCer. A previous report has shown that *Leishmania donovani*-infected macrophages and DCs failed to activate iNKT cells in response to α-GalCer (51). The explanation provided was that *L.donovani* infection increased membrane fluidity and disrupted lipid rafts, which caused the relocation of CD1d molecules from lipid raft-rich to non-lipid raft regions, thus impairing antigen presentation to iNKT cells (51). Herein, we did not observe significant modifications in the expression of CD1d molecules by BM-DC incubated with Exo, EV, or VDE fractions (Figure S4 in Supplementary Material) indicating that these *L. infantum* compounds might use other means to impair CD1d-dependent iNKT cell activation. A similar mechanism leading to the inhibition of iNKT cell activation was also observed with Globotriaosylceramide (Gb3), a glycosphingolipid that accumulates in patients affected by the Fabry disease, a lysosomal storage disorder (52, 53).

It is important to note that lipids extracted from Exo, VDE, and EV mimicked the inhibition of iNKT cell activation induced by the whole fractions. According to previous studies, two main glycocalyx components of *L. donovani*, LPG, and glycoinositolphospholipids (GIPLs), could be presented by CD1d molecules and activate iNKT cells (39, 51, 54). Similar to our findings, LPG and GIPLs could compete with α-GalCer for binding to CD1d molecules (39). *L. donovani* LPG induced CD1d-dependent IFNγ production by iNKT cells (39), in contrast with our findings that revealed no IFNγ, IL-4, or IL-2 production by iNKT cells stimulated with *L. infantum* Exo, VDE, and EV pulsed BM-DC (Figures S3 and S4 in Supplementary Material). The authors hypothesized (39) that iNKT cell activation by *L. donovani* LPG could contribute significantly to resistance in visceral leishmaniasis. We propose that *L. infantum* Exo, VDE, and EV fractions facilitate parasite evasion by impairing iNKT cell proliferation and activation. A possible explanation for these differences is that *Leishmania* LPG and GIPLs present an important polymorphism between the species (55). Another possibility is that molecules other than LPG and GIPLs are responsible for the effect we observed. Consequently, additional studies are required to better characterize the major lipid(s) implicated in this inhibitory effect and determine their molecular mechanism of action. It is possible that *L. infantum* lipid*s* compete with the α-GalCer for the same position within the CD1d binding groove. In this case, they would behave like iNKT cell antagonists since they did not induce cytokine production by iNKT cells. Our results exclude the possibility that parasite products interact with α-GalCer itself to prevent its binding to CD1d since the inhibition of iNKT cells was maintained when plate-bound CD1d were first incubated with Exo, EV, and VDE, washed, and then incubated with α-GalCer. Alternatively, *L. infantum* lipids could eventually interfere with α-GalCer presentation and iNKT cell activation by binding to an external site of the CD1d molecule.

In conclusion, we provide evidence that *L. infantum* exoproducts impair TCR-dependent human iNKT cell expansion and modulate their immune responses. Our results support the conclusion that *L. infantum* Exo, EV, and VDE fractions should be considered as parasite virulence factors that can downregulate protective immunity. Therefore, a better understanding of their contribution to *Leishmania* infection spread and of the regulatory functions of iNKT cells may lead to advances toward the development of an efficient vaccine design.

## Ethics Statement

Experiments using PBMCs from healthy donors were performed in accordance with the Helsinki Declaration, with informed written consent received from each donor and managed by each institution [Etablissement Français du Sang (EFS), Paris, France and Immuno-haemotherapy Department of Hospital de São João, Porto, Portugal]. All experiments were approved by the IBMC.INEB (Instituto de Biologia Molecular e Celular—Instituto de Engenharia Biomédica) Animal Ethics Committees and the Portuguese National Authorities for Animal Health guidelines (directive 2010/63/EU). Begoña Pérez-Cabezas and Anabela Cordeiro-da-Silva are accredited for animal research (Portuguese Veterinary Direction, Ministerial Directive 113/2013).

## Author Contributions

Conceived and designed the experiments: FM, ML-d-M, and AC-d-S. Performed the experiments: RB, NS, and CP. Analyzed the data: RB, NS, CP, FM, BP-C, ML-d-M, and AC-d-S. Contributed with reagents/materials/analysis tools: FM. Wrote the paper: RB, ML-d-M, and AC-d-S.

## Conflict of Interest Statement

The authors declare that the research was conducted in the absence of any commercial or financial relationships that could be construed as a potential conflict of interest.

## Supplementary Material

Figure S1 *Leishmania infantum* Exo, EV, and VDE preparation schema.

Figure S2 *Leishmania infantum* Exo, EV, and VDE alone did not modify iNKT cell function. Human iNKT cell lines were incubated with CD1d-transfected C1R cells alone (−) or previously loaded with α-GalCer 5 ng/ml (+) or *L. infantum* Exo, EV, VDE, or hEV. iNKT cell activation was measured by assessing GM-CSF concentration in culture supernatant. Data show means ± SEM, and the results are representative of two independent experiments (*n* = 4). All groups were tested versus the negative (−) control group (***p* < 0.01).

Figure S3 *Leishmania infantum* Exo, EV, and VDE alone were unable to influence IL-4 and IFNγ production by iNKT cells. Histograms represent the percentage of IL-4^+^ (top panel) or IFNγ^+^ (bottom panel) among iNKT cell line following stimulation by solely CD1d-transfected C1R cells (−) or previously loaded with α-GalCer 5 (+) or *L. infantum* Exo, EV, VDE, or hEV. Data show means ± SEM, and the results are representative of three independent experiments. All groups were tested versus positive (−) control group (***p* < 0.01).

Figure S4 *Leishmania infantum* Exo, EV, and VDE alone failed to inhibit iNKT cell activation in a CD1d dose-dependent manner. **(A)** Plate-bound mouse CD1d was loaded with a mixture of α-GalCer 25 and 100 ng/ml or *L. infantum* Exo, EV, VDE, or hEV at distinct doses. 24.8 iNKT cell hybridoma was added, and supernatants were recovered 20 h later. Data are expressed as means ± SEM of IL-2 levels detected in culture supernatants. Data are representative of two independent experiments (*n* = 4). All groups were tested versus negative control (−) group (***p* < 0.01). **(B)** Representative FACS profile showing the expression of CD1d at the surface of BM-DC previously incubated with α-GalCer alone (medium) or associated with *L. infantum* Exo, EV, VDE, or hEV. Data are representative of three independent experiments.
