# Supplementary figures and images for: Leishmania infantum Exoproducts Inhibit Human Invariant NKT Cell Expansion and Activation
Source: Front Immunol. 2017 Jun 19;8:710. doi: 10.3389/fimmu.2017.00710 (PMC5474685; doi:10.3389/fimmu.2017.00710)

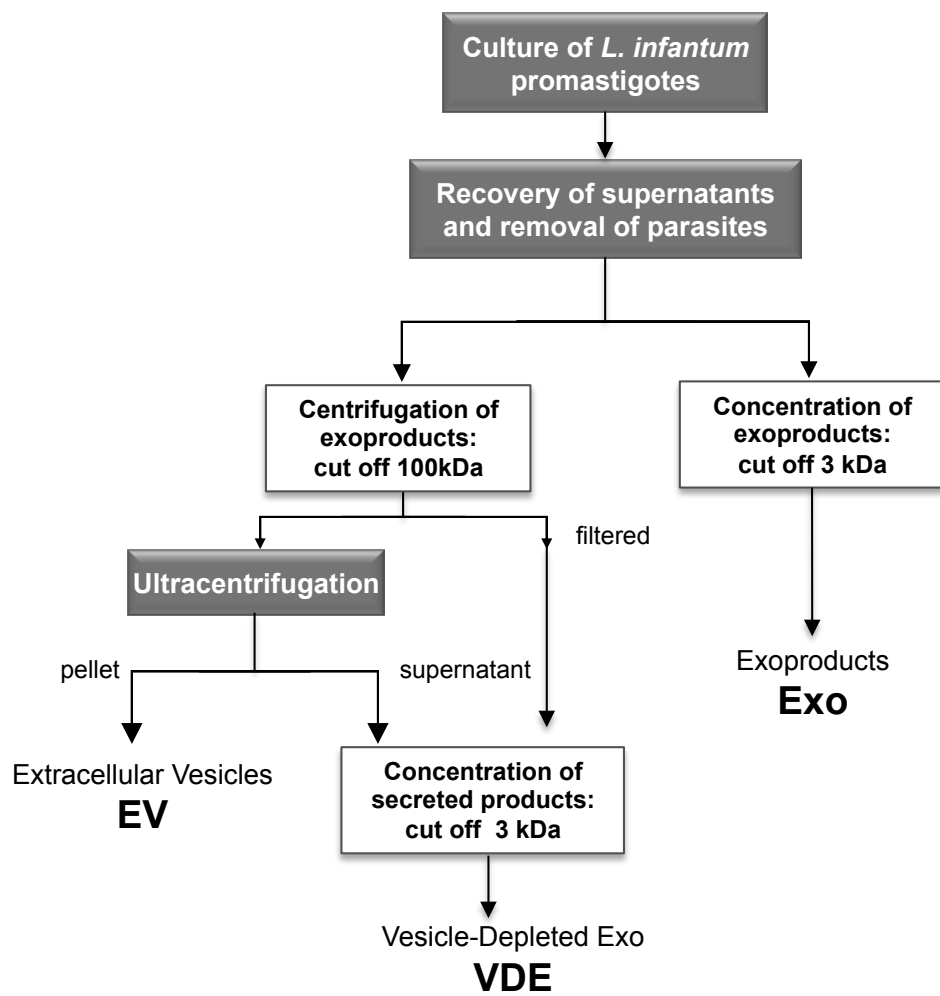

S1 Fig

Supplement: Figure S1 — Leishmania infantum Exo, EV, and VDE preparation schema. [file image_1.pdf]

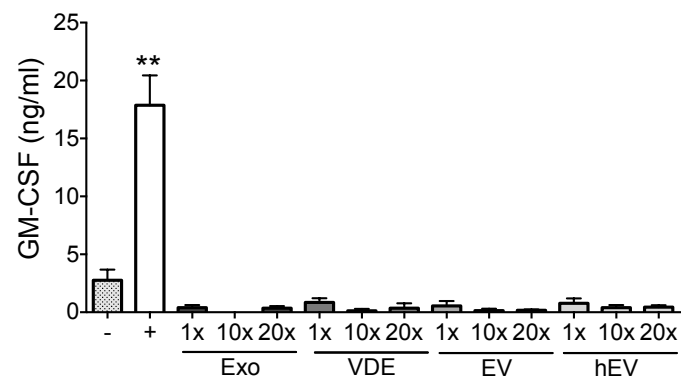

S2 Fig

Supplement: Figure S2 — Leishmania infantum Exo, EV, and VDE alone did not modify iNKT cell function. Human iNKT cell lines were incubated with CD1d-transfected C1R cells alone (−) or previously loaded with α-GalCer 5 ng/ml (+) or L. infantum Exo, EV, VDE, or hEV. iNKT cell activation was measured by assessing GM-CSF concentration in culture supernatant. Data show means ± SEM, and the results are representative of two independent experiments (n = 4). All groups were tested versus the negative (−) control group (**p < 0.01). [file image_2.pdf]

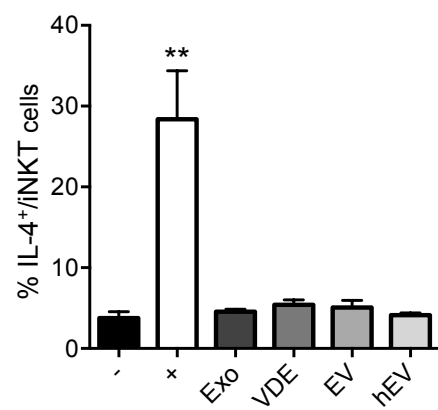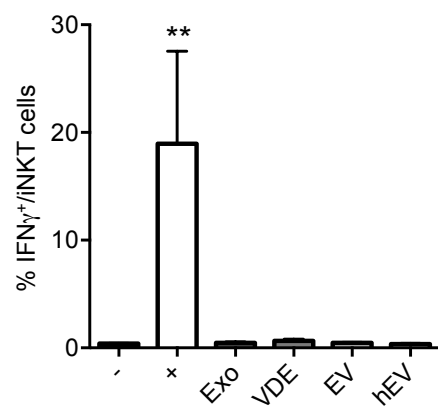

S3 Fig

Supplement: Figure S3 — Leishmania infantum Exo, EV, and VDE alone were unable to influence IL-4 and IFNγ production by iNKT cells. Histograms represent the percentage of IL-4+ (top panel) or IFNγ+ (bottom panel) among iNKT cell line following stimulation by solely CD1d-transfected C1R cells (−) or previously loaded with α-GalCer 5 (+) or L. infantum Exo, EV, VDE, or hEV. Data show means ± SEM, and the results are representative of three independent experiments. All groups were tested versus positive (−) control group (**p < 0.01). [file image_3.pdf]

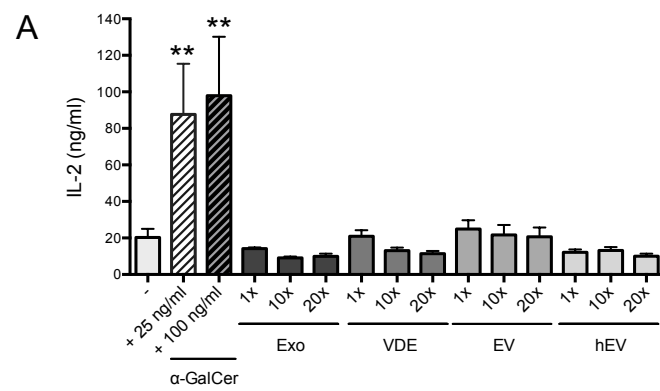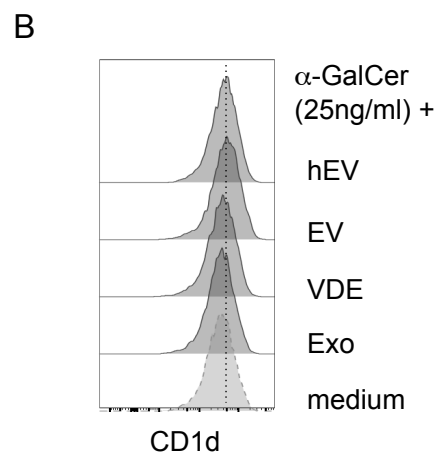

S4 Fig

Supplement: Figure S4 — Leishmania infantum Exo, EV, and VDE alone failed to inhibit iNKT cell activation in a CD1d dose-dependent manner. (A) Plate-bound mouse CD1d was loaded with a mixture of α-GalCer 25 and 100 ng/ml or L. infantum Exo, EV, VDE, or hEV at distinct doses. 24.8 iNKT cell hybridoma was added, and supernatants were recovered 20 h later. Data are expressed as means ± SEM of IL-2 levels detected in culture supernatants. Data are representative of two independent experiments (n = 4). All groups were tested versus negative control (−) group (**p < 0.01). (B) Representative FACS profile showing the expression of CD1d at the surface of BM-DC previously incubated with α-GalCer alone (medium) or associated with L. infantum Exo, EV, VDE, or hEV. Data are representative of three independent experiments. [file image_4.pdf]
